# Familial Mediterranean Fever; Recent Advances, Future Prospectives

**DOI:** 10.3390/diagnostics15070813

**Published:** 2025-03-23

**Authors:** Micol Romano, David Piskin, Ovgu Kul Cinar, Erdal Sag

**Affiliations:** 1 Pediatric Rheumatology, Schulich School of Medicine, Western University, London, ON N6A 3K7, Canada; 2 Canadian Behcet and Autoinflammatory Disease Center (CAN BE AID), Western University, London, ON N6A 3K7, Canada; 3 Department of Epidemiology and Biostatistics, Schulich School of Medicine & Dentistry, University of Western Ontario, London, ON N6A 3K7, Canada; 4 Department of Paediatric Rheumatology, Great Ormond Street Hospital for Children NHS Foundation Trust, Great Ormond Street, London WC1N 3JH, UK; 5 NIHR Biomedical Research Centre, Great Ormond Street Hospital, London WC1N 3BH, UK; 6 Division of Pediatric Rheumatology, Department of Pediatrics, Hacettepe University, 06230 Ankara, Turkey

**Keywords:** Familial Mediterranean Fever, autoinflammation, classification, colchicine, Interleukin-1 inhibitor

## Abstract

Familial Mediterranean Fever (FMF) is the prototype and most common autoinflammatory disease that is particularly frequent in populations originating from the Mediterranean basin. It is characterized by episodes of recurrent inflammation lasting 2–3 days. Colchicine is the mainstay therapy, which decreases the number of attacks and eventually prevents amyloidosis, the most worrisome complication of uncontrolled FMF. It is an autosomal recessive disease. The high rate of *MEFV* gene mutations in specific populations has been discussed as the result of an evolutionary advantage. Tel-Hashomer criteria were the first set of criteria primarily designed for adults. Recently, the Eurofever/PRINTO group has validated a new set of classification criteria for FMF, including clinical and genetic variables. Colchicine intolerance is an important problem and limits the ability to reach an effective dose. In these groups of patients, adding an alternative biological treatment (anti IL-1 agents) is recommended. Several tools such as FMF50, AIDAI, ADDI, ISSF and MASIF have been proposed to evaluate and quantify the disease activity and organ damage. Ongoing research should clarify the exact mechanisms causing FMF attacks and phenotypic variabilities between the patients; further translational research requires the implementation of proteomics and epigenetics signatures to elucidate the pathogenesis.

## 1. Introduction

Familial Mediterranean Fever (FMF) is the most prevalent condition among autoinflammatory diseases, characterized by recurrent fever episodes lasting no more than 3 days. Other typical manifestations include abdominal pain, chest pain, arthritis, and erysipelas-like erythema, commonly seen in lower extremities [1]. FMF is frequently seen in Turkish people, Jewish people, and Armenian people. It is a monogenic autoinflammatory condition caused by autosomal recessive mutations in the *MEFV* gene, which encodes the pyrin (marenostrin) protein [2,3]. Mutations in the *MEFV* gene cause IL-1b overproduction, which in turn results in excessive inflammation. Since 1997, when the *MEFV* gene was identified, many questions have arisen about the impact of these mutations. Although it is an autosomal recessive disease, some patients with a single heterozygous mutation may have the clinical phenotype, though this is still a subject of debate [4,5]. Colchicine is the first-line treatment option for reducing the number of episodes and eventually prevents amyloidosis, the most catastrophic morbidity of FMF. On the other hand, colchicine intolerance or resistance was reported in 5–10% of the patients [6]. Anti-IL1 treatment options were designed due to the pathogenesis of FMF and have since been used as satisfactory options to treat colchicine-resistant/-intolerant patients.

In this review, we evaluated recent advances in the pathogenesis, diagnosis, management, and monitoring of FMF patients and summarized recent evidence-based recommendations.

## 2. Pathogenesis

**Figure 1:**
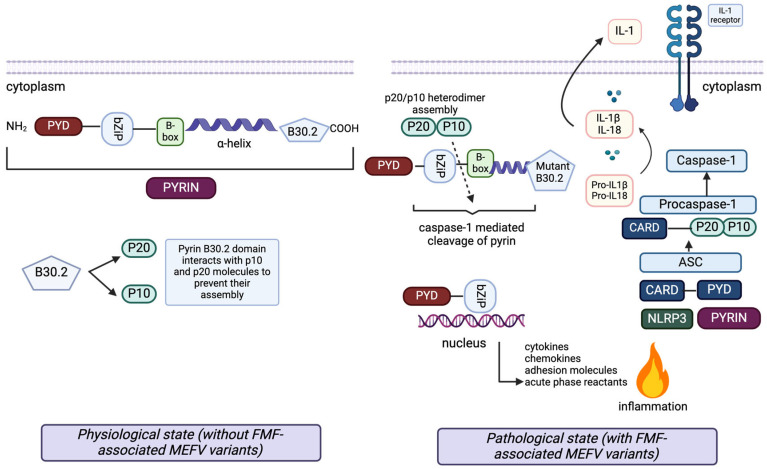
The pathophysiology of the inflammatory response in FMF and the structure of pyrin with its interacting proteins. Pyrin (top left) consists of five domains: the Pyrin domain (PYD), bZip transcription factor basic domain (bZip), B-box zinc finger domain, alpha-helical (coiled coil) domain and B30.2 domain. Each domain has a regulatory role in diverse protein–protein interactions of pyrin with proteins connected to the inflammatory pathways through cell death and apoptosis, secretion of cytokines, chemokines, and adhesion molecules, and cytoskeletal signaling. In the inflammasome (bottom right), active forms of caspase-1 subunits, p20 and p10, are produced. In the cases without any FMF-associated *MEFV* mutations, the pyrin B30.2 domain interacts with p20 and p10 molecules, preventing their assembly into active p20/p10 heterodimers, which activate the caspase-1 mediated cleavage of pyrin. The caspase-1 mediated cleavage site of pyrin is located between the bZip transcription factor basic domain and the B-box zinc finger domain.

FMF is inherited with autosomal recessive mutations on the *MEFV* gene, which encodes the pyrin protein [2,3]. Pyrin is a fundamental element of the NLRP3 inflammasome complex. Mutation of this protein initiates caspase-1 activation and IL-1b production, causing excessive inflammation (Figure 1) [7].

Additionally, the wild-type B30.2 domain of pyrin helps to create a blockade for IL-1β secretion by interacting with proIL-1β. In the presence of FMF-associated *MEFV* mutations, the mutant B30.2 pyrin domain shows reduced interaction with p20 and p10, thereby allowing p20/p10 heterodimer assembly (active caspase-1), cleavage of pyrin, IL-1β activation and induction of inflammation (left). The N-terminal cleaved fragment of pyrin interacts with IκB-α, p65 and p50 molecules through the bZip domain. Subsequently, it moves into the nucleus to enhance the activation of the NF-κB transcription factor and expression of the proinflammatory genes (bottom).

Chae et al. have shown in *MEFV* knock-in mice models that gain-of-function mutations of pyrin protein led to overproduction of IL-1b via associated speck-like protein-containing CARD (ASC) and caspase-1 activation [8]. Furthermore, pyrin mediates caspase-1 activation in response to pathogen modification and inactivation of RhoA-GTPases by several bacterial toxins [9]. There is a control mechanism for pyrin inflammasome in healthy individuals. RhoA-activated serine-threonine kinases (PKN1 and PKN 2) bind and phosphorylate pyrin, which binds to regulatory 14-3-3 proteins, inhibiting pyrin inflammasome. Most of the common and severe *MEFV* mutations are clustered around the C terminal of the B30.2 domain, which is important for controlling pyrin phosphorylation by inhibiting the binding of PKN1, resulting in a lowered threshold for pyrin inflammasome activation. In parallel with this finding, it was shown that colchicine both activates RhoA and reverses the inhibition of RhoA by bacterial toxins [10].

A recent study by Park et al. has reported the possible selective advantage of *MEFV* mutations, since their carrier frequencies are very high in several Mediterranean populations. They have found that mutated pyrin interacts less with *Yersinia pestis* virulence factor YopM, attenuating YopM-induced IL-1b suppression. This was proven in vitro and during in vivo studies, as well. Leucocytes from patients with homozygous or compound heterozygous and asymptomatic heterozygous carriers released more IL-1b in response to *Y. pestis* than healthy controls. Similarly, *Y. pestis*-infected *MEFV*^M680I/M680I^ FMF knock-in mice had IL-1-dependent increased survival relative to wild-type knock-in mice. In summary, FMF mutations in Mediterranean populations might lead to a positive selection against plague due to increased resistance to *Y. pestis* [11].

## 3. Diagnosis and Classification

A diagnosis of FMF relies on characteristic clinical features, and the presence of *MEFV* mutation usually supports this diagnosis. Various attempts have been made to classify FMF since 1997 (Table 1).

The first criteria set for FMF was proposed mainly for adults and these criteria are referred to as Tel-Hashomer criteria [12]. Since this set of criteria is designed primarily for adults, and there are some differences between adults and children, Turkish FMF pediatric criteria, also known as Yalcinkaya-Ozen criteria, have been proposed [13]. Both these criteria sets are composed of clinical features only. They do not include the genetic variants or ethnicity data overall. Recently, the Eurofever/PRINTO group published new classification criteria, including a combination of ethnicity, clinical manifestations and genotype [14]. This new classification criteria offers two options for detecting genetic variants. The care physician can make a diagnosis based on ≥6/9 clinical criteria if *MEFV* variant analysis is not feasible. On the other hand, according to the *MEFV* genotype, one or two clinical criteria (out of four criteria) would be sufficient if genetic testing is possible. Among Turkish children, Eurofever/PRINTO criteria were the most sensitive (96% vs. 89% Tel-Hashomer; 93% Yalcinkaya-Ozen), but the least specific (73% vs. 93% Tel-Hashomer; 84% Yalcinkaya-Ozen) among these three criteria sets [15]. The combination of clinical findings and genetic testing increased the sensitivity. Although the ethnicity criteria seem to lower the specificity of the newest criteria set, having the “clinical-only” option may provide guidance which helps the physician to choose and order relevant genetic tests for patients, especially in non-endemic countries. Since 1997, the discovery of the link between the *MEFV* gene and FMF has increased the number of variants gradually, causing problems when it comes to deciding which variants are pathogenic. Phenotype–genotype descriptions related to the different *MEFV* variants are summarized and recorded in the INFEVERS database (https://infevers.umai-montpellier.fr/web/search.php?n=1 (accessed on 12 January 2025)), in which there are 401 known variants to date. These variants have been graded by an expert panel as pathogenic, likely pathogenic, benign, likely benign, and a variant of unknown significance (VUS) [16]. Screening for these 401 variants is not practical. Disease-causing pathogenic variants are commonly located in exon 10 (M694V, V726A, M680I and M694I). E148Q, which is in exon two, is the most frequent VUS in carriers. To provide clinicians, scientists, and geneticists with guidance on appropriate molecular genetic testing, an international task force developed recommendations and proposed screening a total of 14 variants for genetic confirmation of the patients (pathogenic variants: M694V, M694I, M680I, V726A, R761H, A744S, E167D, T267I and I692del VUS: K695R, E148Q, P369S, F479L, and I591T) [17]. In various studies, exon 10 mutations, specifically M694V mutations, are associated with early-onset disease with the more severe course and more frequently associated with other inflammatory comorbidities such as Behçet’s disease, inflammatory bowel diseases, vasculitis, etc. [18,19,20]. Although E148Q variants are reported as VUS and heterozygous E148Q mutations do not support the diagnosis of FMF according to the recent recommendations, in a recent study involving 169 patients (amyloidosis secondary to FMF), E148Q homozygous mutations were reported in 19 patients. In another study, 7 out of 22 FMF patients with phenotype 2 had variants in the E148Q, which was a higher number than expected [17,20,21,22]. Up-to-date recommendations for the genetic diagnosis of FMF are in Table 2.

These recommendations are supported by “The ISAAID/EMQN Best Practice Guidelines for the genetic diagnosis of monogenic autoinflammatory disease”. These guidelines also recommend confirming the diagnosis if there are biallelic pathogenic variants. However, the decision is left to the expert clinicians if there is either one pathogenic variant + one VUS, only one pathogenic variant, or biallelic VUS mutations [24].

Although FMF is an autosomal recessive disease, some patients with heterozygous mutations also presented with typical FMF clinical features. It was reported that approximately 25% of the clinically confirmed FMF patients did not have a biallelic mutation in the *MEFV* gene [17]. Previously, it was thought that routine sequencing techniques had missed less common variants. However, Booty et al. [25] failed to define any intragenic genomic mutations in 46 heterozygous FMF patients with new techniques, and Marek-Yagel et al. [26] failed to define any further mutations or abnormal methylation patterns via sequencing complementary DNA in heterozygote FMF patients. On the other hand, in a recent study of 46 patients with undefined autoinflammatory diseases, a new method using an NGS-based multiplex array enabling a “long-amplicon” approach defined 46 nonsynonymous variations, 169 synonymous variations, 89 UTR variations, and 588 intronic variations in the *MEFV* gene, which proves that deep screening may still help to define some modifications in these groups of patients [27]. One possible explanation for carriers presenting with typical clinical features might be the effect of modifier genes related to inflammation. Certain polymorphisms in the innate immune system genes, *SAA* and *TLR2*, were shown to be linked to the development of amyloidosis [28,29,30]. Thus, if certain polymorphisms may affect the disease course, they could be the causative mechanism in heterozygote FMF patients with typical FMF phenotype [31,32].

## 4. Treatment

Colchicine has been the first line of treatment for FMF since 1972 [33]. It reduces the frequency of episodes and disease severity, improves quality of life, and prevents the most devastating complication, amyloidosis [34,35]. The suggested colchicine dosage for children less than five years old is ≤0.5 mg/day (≤0.6 mg/day in tablets containing 0.6 mg) and for children between five and ten years of age, it is 0.5–1.0 mg/day (1.2 mg/day if the tablets contain 0.6 mg). The recommended colchicine dosage for the pediatric age group older than 10 years and the adult population is 1.0–1.5 mg/day (1.8 mg/day if tablets contain 0.6 mg).

The maximum colchicine dosage should not exceed 3 mg/day for adults and 2 mg/day for children [6,23,36]. Historically, it was generally administered twice daily; Polat et al. showed that once-daily usage is not inferior to twice daily usage in their randomized controlled non-inferiority trial [37]. Patients should be followed at least every 6 months for disease activity, treatment adherence, and side effects. Approximately 5–10% of the patients are non-responsive or intolerant to colchicine. Recently, an expert panel reported the consensus-based definition of colchicine resistance and intolerance. Colchicine resistance was described as (if a patient is on the maximum tolerated colchicine dose) ongoing disease activity defined as recurrent clinical episodes or persistent elevation of inflammatory markers (SAA or CRP) in between episodes without any other reason [6]. Intolerance of colchicine in patients with FMF mainly presents in the form of gastrointestinal symptoms, including diarrhea and nausea, which causes them to be unable to reach the desired or effective dosage. Colchicine toxicity is different from intolerance and is described as severe gastrointestinal involvement, bone marrow suppression, and neuromyopathy [6]. In these groups of patients, adding an alternative biological treatment is recommended. Due to the pathogenesis of FMF, pyrin inflammasome results in increased IL-1β production. Thus, anti-IL1 agents are preferred as the first-line biological treatment [23].

Anti-IL-1 therapies, including Anakinra (an IL-1 receptor antagonist) and Canakinumab (an anti-IL-1β monoclonal antibody), competitively inhibit IL-1R1 or neutralize IL-1β, thereby reducing proinflammatory cytokine production and neutrophil infiltration. These mechanisms collectively demonstrate the clinical effectiveness of IL-1 blockade in managing systemic inflammation. Anakinra and Canakinumab were reported in many publications, including six randomized controlled trials (one anakinra, four canakinumab and one rilonacept). Despite the marked heterogeneity in outcome measures across studies, the majority prioritized reductions in acute-phase reactants and attack frequency as primary endpoints, with all anti-IL-1 agents demonstrating consistent efficacy in these parameters. They demonstrated a favorable safety profile, with predominantly mild adverse events limited to injection site reactions, uncomplicated infections, transient elevations in liver enzymes, and self-limiting leukopenia [38,39,40,41,42,43]. There are also two randomized controlled trials with anti-IL6 treatment (tocilizumab). Despite both RCTs failing to achieve the primary endpoint, anti-IL6 treatments appear to be successful in reducing the frequency of attacks and acute-phase reactants while also enhancing various disease activity scores and patient-reported outcomes [44,45,46].

In recent studies, JAK1/TYK2 (which act downstream of the IFN receptor) were found to be essential for regulating IL-1β-mediated inflammation by network analyses and in silico tests supported by the fact that pyrin and gasdermin-D are interferon-inducible genes [47]. Emerging clinical evidence complements preclinical findings, with three independent case series involving six FMF patients reporting that tofacitinib—a Janus kinase (JAK) inhibitor—demonstrated marked suppression of inflammatory biomarkers and achieved sustained clinical remission [48,49,50]. These observations, supported by a mechanistic rationale implicating JAK-STAT signaling in FMF pathogenesis, position tofacitinib as a promising therapeutic candidate warranting further investigation in larger controlled trials.

The most recent recommendations for the management and follow-up of FMF patients are given in Table 2.

## 5. Outcome-Measuring Tools

In parallel with a quote from economics, ‘If you cannot measure it, you cannot manage it’, physicians still debate the definition and concept of disease severity, activity, and damage. However, the ability to collect standardized data will significantly improve the care of patients with FMF.

### 5.1. Disease Activity

The AutoInflammatory Disease Activity Index (AIDAI) was designed to evaluate active/inactive disease for the four major hereditary recurrent fever syndromes, including FMF. It comprises 13 items, including fever, overall symptoms, abdominal pain, nausea/vomiting, diarrhea, headaches, chest pain, painful nodes, arthralgia, or myalgia, swelling of the joints, eye manifestations, skin rash, and pain relief. This daily diary allows patients or parents to record their score as the presence or absence of the symptom (yes (1 point) or no (0 point)). In order to differentiate active disease from inactive disease, AIDAI researchers defined a cutoff score of ≥9 as having a specificity of 92% and sensitivity of 89% [51]. This score was externally validated with the colchicine-resistant FMF patients in the phase III CLUSTER trial, as well. The researchers found that AIDAI scores ≥ 12 were the optimal threshold to discriminate inactive disease with a specificity of 79% and sensitivity of 75% [52]. On the other hand, a recent study on 239 FMF patients showed that the number of episodes, rather than AIDAI, was the only key element that can be used as a valid indicator of disease activity [53].

### 5.2. Disease Severity

The International Severity Score for FMF (ISSF) was developed to assess the disease severity, composed of 10 different items including chronic sequela, organ dysfunction, organ failure, frequency of attacks, increased acute-phase reactants during attack-free periods, involvement of more than two sites, more than two different types of attack, duration of attacks, and exertional leg pain. According to this scoring system, patients were stratified as having severe disease (ISSF score ≥ 6), intermediate disease (ISSF score: 3–5), or mild disease (ISSF score ≤ 2) [54].

### 5.3. Disease Damage

The Autoinflammatory Disease Damage Index (ADDI) was developed to measure the damage caused by autoinflammatory diseases as an outcome parameter. It contains 18 items sub-categorized into eight organ systems (reproductive, renal/amyloidosis, developmental, serosal, neurological, ears, ocular and musculoskeletal damage) with different score weights. Damage was defined as “*a persistent or irreversible change in structure or function that was present for at least 6 months*” [55]. This score was validated by 37 experts on 110 paper clinical cases, revealing that it was strongly correlated with Physician Global Assessment of Damage and was not strongly influenced by disease activity [56].

### 5.4. Assessment of Treatment Outcome Indices

The FMF50 score was developed by the Turkish FMF Study group and FAVOR (FMF Arthritis Vasculitis and Orphan disease Research in pediatric rheumatology) to assess the response of the colchicine treatment. It consisted of six criteria: 1. percentage change in the frequency of attacks with the treatment; 2. percentage change in the duration of attacks with the treatment; 3. patients/parents’ global assessment of disease severity (10 cm visual analog scale (VAS)); 4. physicians’ global assessment of disease severity (10 cm VAS); 5. percentage change in arthritis attacks with the treatment; and 6. percentage change in C-reactive protein, erythrocyte sedimentation rate, or serum amyloid A level with the treatment. The response to treatment was determined to be at least a 50% improvement in five of six items among the criteria set, without worsening in any criterion, according to the original study [57]. Nonetheless, this score requires prospective validation in different cohorts to be used more widely.

The Medication Adherence Scale in Familial Mediterranean Fever (MASIF) was developed to evaluate adherence to the treatment. It comprises 18 items clustered in four domains, including knowledge about the medication, adherence to the treatment, barriers to drug use, and factors that may increase compliance. Each of these 18 items was scored 1–5 (1: strongly agree; 5: strongly disagree), resulting in a total score ranging from 18 to 90. Patients were deemed to have good medication adherence if their score was over 60 [58].

In conclusion, the availability of these scores for clinical practice and clinical trials may facilitate the long-term management of individual patients. To guide clinicians, we recommend using AIDAI and MASIF as patient-reported instruments. The AIDAI is a valid and reliable patient diary used to assess disease activity. It has been suggested that at least a three-month diary is required to decide on disease activity. However, this can be challenging in patients who experience fewer episodes during the year. In addition, MASIF may be a useful tool for identifying colchicine-resistant patients with the ability to differentiate poor adherence from nonresponse to colchicine. FMF50 is a well-performing index for measuring response to treatment in all drug studies for colchicine-resistant cases. Finally, ISSF is the most detailed tool for measuring severity scores for FMF. Close monitoring of patients with severe disease may protect them from poor prognosis.

## 6. Future Perspectives

Despite well-identified triggers, the exact mechanisms causing FMF attacks need better elucidation. For instance, the typical characteristics of attacks, which usually last 48–72 h followed by spontaneous resolution, remain unknown. It is still not clear why some carriers present with clinical symptoms. FMF has large phenotypic diversity even in patients with the same genotype. Identifying the factors contributing to disease variability is essential, as it will help us to understand this condition better. Epigenetic changes in *MEFV* have been suggested as a potential modifier of the pathogenic effect of disease-causing *MEFV* mutations and may explain different phenotypes or severity. This critical knowledge gap also needs to be addressed with further studies. The possible association between FMF and other diseases should be explored. For instance, patients with homozygous exon 10 mutations are more prone to sacroiliitis, vasculitis, and inflammatory bowel diseases. However, patients with less pathogenic variants or heterozygous mutations may also display these comorbidities. Further translational studies, including multi-omics approaches, are needed to shed light on these matters.

Moreover, biomarkers are needed to monitor disease progression and treatment response. Recently, Koga et al. demonstrated a significant decrease in serum CXCL1 and VEGF levels in colchicine-resistant FMF patients receiving tocilizumab treatment [59]. Therefore, levels of CXCL1 and VEGF may be used as potential biomarkers for elucidating the inflammatory state and disease progression. Further large-scale longitudinal studies with repeated measures are required to confirm the role of CRP, SAA, and S100 proteins and these recently described potential biomarkers.

The reason why FMF-related AA amyloidosis has a poor prognosis is not fully understood. One possible explanation is that once amyloid deposition starts, it causes damage, such as fibrosis, sclerosis, and kidney injury. It can continue to worsen even if the amyloid deposition stops. A recent study showed that most patients with FMF amyloidosis will experience kidney failure or die within 11 years of being diagnosed, and using IL-1 blockers after diagnosis of amyloidosis does not appear to improve outcomes [60]. Two studies from Turkey analyzed the efficacy of tocilizumab in 27 adult patients with FMF-related AA amyloidosis [61,62]. In most cases, tocilizumab effectively slowed the progression of amyloidosis and controlled the disease activity. These data confirmed the hypothesis that IL-6 inhibition is mainly effective in FMF patients with renal amyloidosis. However, further prospective studies are needed to determine if IL-1 or IL-6 blockers could be useful in improving outcomes for patients with FMF-related AA amyloidosis.

The role of *anakinra in on-demand* administration remains unclear. A recent article suggests that a single dose of anakinra effectively prevents disease episodes within 24 h and shortens the duration of hospitalization [63]. Future clinical trials are required to confirm these findings.

Overall, expanding and improving prospective longitudinal registries with the implementation of biobanking will contribute to research efforts aiming to validate biomarkers for monitoring, improve patient outcomes, and ultimately discover novel treatment strategies.

## Figures and Tables

**Table 1:** The different classification criteria of FMF.

Tel-Hashomer Criteria [12]	Yalcinkaya-Ozen Criteria [13]	Eurofever/PRINTO [14]	
		Clinical-Only Criteria	Clinical + Genetic Criteria
**Major criteria** Recurrent febrile episodes with serositis (peritonitis, synovitis or pleuritis) Amyloidosis of AA type without a predisposing disease Favorable response to regular colchicine treatment **Minor criteria** Recurrent febrile episodes FMF in a first-degree relative Erysipelas-like erythema ≥2 major or 1 major + 2 minor criteria	Fever (Axillary temperature of >38 °C, 6–72 h of duration, ≥3 attacks) Abdominal pain (6–72 h of duration, ≥3 attacks) Chest pain (6–72 h of duration, ≥3 attacks) Arthritis (6–72 h of duration, ≥3 attacks, oligoarthritis) Family history of FMF ≥2 criteria	**Presence of** Eastern Mediterranean ethnicity Duration of episodes 1–3 days Arthritis Chest pain Abdominal pain **Absence of** Aphthous stomatitis Urticarial rash Maculopapular rash Painful lymph nodes ≥6 criteria	**Presence of confirmatory *MEFV*** genotype and **at** **least one** among the following: Duration of episodes 1–3 days Arthritis Chest pain Abdominal pain **ORPresence** **of not confirmatory *MEFV*** genotype and **at least two** among the following: Duration of episodes 1–3 days 2. Arthritis 3. Chest pain 4. Abdominal pain

**Table 2:** Recommendations about diagnosis, treatment, and management of FMF patients [20,23].

Diagnosis
FMF is a clinical diagnosis which can be supported, but not excluded, by genetic testing. Consider patients homozygous for *M694V* at risk of developing, with very high probability, a severe phenotype. FMF patients carrying two of the common mutated alleles (homozygotes or compound heterozygotes), especially for *M694V* mutation or mutations at position 680–694 on exon 10, must be considered at risk of more severe disease. The E148Q variant is common, of unknown pathogenic significance, and, as the only *MEFV* variant, does not support the diagnosis of FMF. Patients homozygous for M694V mutation are at risk of early-onset disease. Asymptomatic individuals with homozygous M694V variants should be evaluated and followed closely to consider therapy. For individuals with two pathogenic mutations for FMF who do not report symptoms, if there are risk factors for AA amyloidosis (such as the country, family history, and persistently elevated inflammatory markers, particularly serum amyloid A protein), close follow-up should be started, and treatment considered. Consultation with an AID specialist may be helpful to assist with the indication and interpretation of the genetic testing and diagnosis.
**Treatment**
Colchicine treatment should be started as soon as a clinical diagnosis is made. Depending on tolerance and compliance, doses can be single or divided. The persistence of attacks or subclinical inflammation represents an indication to increase the colchicine dose. Compliant patients who are not responding to the maximum tolerated dose of colchicine can be considered non-responders or resistant; alternative biological treatments are indicated in these patients. FMF treatment needs to be intensified in AA amyloidosis using the maximal tolerated dose of colchicine and supplemented with biologics as required. Periods of physical or emotional stress can trigger FMF attacks, and it may be appropriate to increase the dose of colchicine temporarily. When suspecting an attack, other possible causes should always be considered. During attacks, the usual dose of colchicine can be continued, and nonsteroidal anti-inflammatory drugs (NSAIDs) can be administered. Chronic arthritis in a patient with FMF might necessitate additional medications, such as conventional DMARDs, intra-articular steroid injections, or biologics. In protracted febrile myalgia, glucocorticoids may lead to the resolution of symptoms, and NSAIDs and IL-1 blockade might be considered for treatment. In addition, NSAIDs are recommended for the treatment of exertional leg pain.
**Management**
The ultimate goal of treatment in FMF is to achieve complete control of unprovoked attacks and to minimize subclinical inflammation in between attacks. Response, toxicity, and compliance should be monitored every 6 months Liver enzymes should be monitored regularly; if liver enzymes are elevated by more than two-fold the upper limit of normal, colchicine should be reduced, and transaminitis should be investigated. In patients with decreased renal function, the risk of toxicity is very high; therefore, signs of colchicine toxicity, as well as CPK, should be monitored carefully, and the colchicine dose should be reduced accordingly. Colchicine toxicity is a serious complication and should be adequately suspected and prevented. Colchicine should not be discontinued during conception, pregnancy, or lactation; current evidence does not justify amniocentesis. In general, males do not need to stop colchicine before conception; however, a temporary dose reduction or discontinuation may be needed in the rare case of azoospermia or oligospermia proven to be related to colchicine. If a patient is stable, has had no attacks for more than 5 years, and has no elevated APR, dose reduction could be considered after expert consultation and continued monitoring.

## Data Availability

Data are contained within the article.

## Abbreviations

The following abbreviations are used in this manuscript:

FMF	Familial Mediterranean Fever.
YopM	*Yersinia pestis* virulence factor.
VUS	Variance of insignificance.
ISSAID	International Society of Autoinflammatory Diseases.
SAA	Serum amyloid A.
AIDAI	Autoinflammatory Diseases Activity Index.
ADDI	Autoinflammatory Disease Damage Index.
ISSF	International Severity Score for FMF.
MASIF	Medication Adherence Scale in FMF.
JAIMAR	Juvenile Autoinflammatory Disease Multidimensional Assessment Report.
FAVOR	FMF Arthritis Vasculitis and Orphan Disease Research in pediatric rheumatology.
VAS	Visual analog scale.

## References

[1] Sag E., Bilginer Y., Ozen S. (2017). Autoinflammatory Diseases with Periodic Fevers. Curr. Rheumatol. Rep..

[2] French F.M.F.C. (1997). A candidate gene for familial Mediterranean fever. Nat. Genet..

[3] The International FMF Consortium (1997). Ancient missense mutations in a new member of the RoRet gene family are likely to cause familial Mediterranean fever. Cell.

[4] Kalyoncu M., Acar B.C., Cakar N., Bakkaloglu A., Ozturk S., Dereli E., Tunca M., Kasapcopur O., Yalcinkaya F., Ozen S. (2006). Are carriers for MEFV mutations “healthy”?. Clin. Exp. Rheumatol..

[5] Lachmann H.J., Sengul B., Yavuzsen T.U., Booth D.R., Booth S.E., Bybee A., Gallimore J.R., Soyturk M., Akar S., Tunca M. (2006). Clinical and subclinical inflammation in patients with familial Mediterranean fever and in heterozygous carriers of MEFV mutations. Rheumatology.

[6] Ozen S., Sag E., Ben-Chetrit E., Gattorno M., Gul A., Hashkes P.J., Kone-Paut I., Lachmann H.J., Tsitsami E., Twilt M. (2020). Defining colchicine resistance/intolerance in patients with familial Mediterranean fever: A modified-Delphi consensus approach. Rheumatology.

[7] Ozen S., Bilginer Y. (2014). A clinical guide to autoinflammatory diseases: Familial Mediterranean fever and next-of-kin. Nat. Rev. Rheumatol..

[8] Chae J.J., Cho Y.H., Lee G.S., Cheng J., Liu P.P., Feigenbaum L., Katz S.I., Kastner D.L. (2011). Gain-of-function Pyrin mutations induce NLRP3 protein-independent interleukin-1beta activation and severe autoinflammation in mice. Immunity.

[9] Xu H., Yang J., Gao W., Li L., Li P., Zhang L., Gong Y.N., Peng X., Xi J.J., Chen S. (2014). Innate immune sensing of bacterial modifications of Rho GTPases by the Pyrin inflammasome. Nature.

[10] Park Y.H., Wood G., Kastner D.L., Chae J.J. (2016). Pyrin inflammasome activation and RhoA signaling in the autoinflammatory diseases FMF and HIDS. Nat. Immunol..

[11] Park Y.H., Remmers E.F., Lee W., Ombrello A.K., Chung L.K., Shilei Z., Stone D.L., Ivanov M.I., Loeven N.A., Barron K.S. (2020). Ancient familial Mediterranean fever mutations in human pyrin and resistance to *Yersinia pestis*. Nat. Immunol..

[12] Livneh A., Langevitz P., Zemer D., Zaks N., Kees S., Lidar T., Migdal A., Padeh S., Pras M. (1997). Criteria for the diagnosis of familial Mediterranean fever. Arthritis Rheum..

[13] Yalcinkaya F., Ozen S., Ozcakar Z.B., Aktay N., Cakar N., Duzova A., Kasapcopur O., Elhan A.H., Doganay B., Ekim M. (2009). A new set of criteria for the diagnosis of familial Mediterranean fever in childhood. Rheumatology.

[14] Gattorno M., Hofer M., Federici S., Vanoni F., Bovis F., Aksentijevich I., Anton J., Arostegui J.I., Barron K., Ben-Cherit E. (2019). Classification criteria for autoinflammatory recurrent fevers. Ann. Rheum. Dis..

[15] Sag E., Demirel D., Demir S., Atalay E., Akca U., Bilginer Y., Ozen S. (2020). Performance of the new ‘Eurofever/PRINTO classification criteria’ in FMF patients. Semin. Arthritis Rheum..

[16] Van Gijn M.E., Ceccherini I., Shinar Y., Carbo E.C., Slofstra M., Arostegui J.I., Sarrabay G., Rowczenio D., Omoyimni E., Balci-Peynircioglu B. (2018). New workflow for classification of genetic variants’ pathogenicity applied to hereditary recurrent fevers by the International Study Group for Systemic Autoinflammatory Diseases (INSAID). J. Med. Genet..

[17] Shinar Y., Obici L., Aksentijevich I., Bennetts B., Austrup F., Ceccherini I., Costa J.M., De Leener A., Gattorno M., Kania U. (2012). Guidelines for the genetic diagnosis of hereditary recurrent fevers. Ann. Rheum. Dis..

[18] Ozturk K., Coskuner T., Baglan E., Sonmez H.E., Yener G.O., Cakmak F., Demirkan F.G., Tanatar A., Karadag S.G., Ozdel S. (2021). Real-Life Data From the Largest Pediatric Familial Mediterranean Fever Cohort. Front. Pediatr..

[19] Balci-Peynircioglu B., Kaya-Akca U., Arici Z.S., Avci E., Akkaya-Ulum Z.Y., Karadag O., Kalyoncu U., Bilginer Y., Yilmaz E., Ozen S. (2020). Comorbidities in familial Mediterranean fever: Analysis of 2000 genetically confirmed patients. Rheumatology.

[20] Giancane G., Ter Haar N.M., Wulffraat N., Vastert S.J., Barron K., Hentgen V., Kallinich T., Ozdogan H., Anton J., Brogan P. (2015). Evidence-based recommendations for genetic diagnosis of familial Mediterranean fever. Ann. Rheum. Dis..

[21] Arici Z.S., Romano M., Piskin D., Guzel F., Sahin S., Berard R.A., Yilmaz M.I., Demirkaya E. (2021). Evaluation of E148Q and Concomitant AA Amyloidosis in Patients with Familial Mediterranean Fever. J. Clin. Med..

[22] Altunoğlu A., Erten Ş., Canoz M.B., Yuksel A., Ceylan G.G., Balci S., Dogan H.T. (2013). Phenotype 2 familial mediterranean fever: Evaluation of 22 case series and review of the literature on phenotype 2 FMF. Ren. Fail..

[23] Ozen S., Demirkaya E., Erer B., Livneh A., Ben-Chetrit E., Giancane G., Ozdogan H., Abu I., Gattorno M., Hawkins P.N. (2016). EULAR recommendations for the management of familial Mediterranean fever. Ann. Rheum. Dis..

[24] Shinar Y., Ceccherini I., Rowczenio D., Aksentijevich I., Arostegui J., Ben-Chetrit E., Boursier G., Gattorno M., Hayrapetyan H., Ida H. (2020). ISSAID/EMQN Best Practice Guidelines for the Genetic Diagnosis of Monogenic Autoinflammatory Diseases in the Next-Generation Sequencing Era. Clin. Chem..

[25] Booty M.G., Chae J.J., Masters S.L., Remmers E.F., Barham B., Le J.M., Barron K.S., Holland S.M., Kastner D.L., Aksentijevich I. (2009). Familial Mediterranean fever with a single MEFV mutation: Where is the second hit?. Arthritis Rheum..

[26] Marek-Yagel D., Berkun Y., Padeh S., Abu A., Reznik-Wolf H., Livneh A., Pras M., Pras E. (2009). Clinical disease among patients heterozygous for familial Mediterranean fever. Arthritis Rheum..

[27] Guzel F., Romano M., Keles E., Piskin D., Ozen S., Poyrazoglu H., Kasapcopur O., Demirkaya E. (2021). Next Generation Sequencing Based Multiplex Long-Range PCR for Routine Genotyping of Autoinflammatory Disorders. Front. Immunol..

[28] Migita K., Agematsu K., Masumoto J., Ida H., Honda S., Jiuchi Y., Izumi Y., Maeda Y., Uehara R., Nakamura Y. (2013). The contribution of SAA1 polymorphisms to Familial Mediterranean fever susceptibility in the Japanese population. PLoS ONE.

[29] Yilmaz E., Balci B., Kutlay S., Ozen S., Erturk S., Oner A., Besbas N., Bakkaloglu A. (2003). Analysis of the modifying effects of SAA1, SAA2 and TNF-alpha gene polymorphisms on development of amyloidosis in FMF patients. Turk. J. Pediatr..

[30] Soylu A., Ates H., Cingoz S., Turkmen M., Demir B.K., Tunca M., Sakizli M., Cirit M., Ersoy R., Ulgenalp A. (2011). TLR polymorphisms in FMF: Association of TLR-2 (Arg753Gln) and TLR-4 (Asp299Gly, Thre399Ile) polymorphisms and myeloid cell TLR-2 and TLR-4 expression with the development of secondary amyloidosis in FMF. Inflammation.

[31] Touitou I., Picot M.C., Domingo C., Notarnicola C., Cattan D., Demaille J., Koné-Paut I. (2001). The MICA region determines the first modifier locus in familial Mediterranean fever. Arthritis Rheum..

[32] Cazeneuve C., Ajrapetyan H., Papin S., Roudot-Thoraval F., Geneviève D., Mndjoyan E., Papazian M., Sarkisian A., Babloyan A., Boissier B. (2000). Identification of MEFV-independent modifying genetic factors for familial Mediterranean fever. Am. J. Hum. Genet..

[33] Goldfinger S.E. (1972). Colchicine for familial Mediterranean fever. N. Engl. J. Med..

[34] Demirkaya E., Erer B., Ozen S., Ben-Chetrit E. (2016). Efficacy and safety of treatments in Familial Mediterranean fever: A systematic review. Rheumatol. Int..

[35] Livneh A., Zemer D., Langevitz P., Shemer J., Sohar E., Pras M. (1993). Colchicine in the treatment of AA and AL amyloidosis. Semin. Arthritis Rheum..

[36] Kallinich T., Haffner D., Niehues T., Huss K., Lainka E., Neudorf U., Schaefer C., Stojanov S., Timmann C., Keitzer R. (2007). Colchicine use in children and adolescents with familial Mediterranean fever: Literature review and consensus statement. Pediatrics.

[37] Polat A., Acikel C., Sozeri B., Dursun I., Kasapcopur O., Gulez N., Simsek D., Saldir M., Dokurel I., Poyrazoglu H. (2015). Comparison of the efficacy of once- and twice-daily colchicine dosage in pediatric patients with familial Mediterranean fever—A randomized controlled noninferiority trial. Arthritis Res. Ther..

[38] Ben-Zvi I., Kukuy O., Giat E., Pras E., Feld O., Kivity S., Perski O., Bornstein G., Grossman C., Harari G. (2017). Anakinra for Colchicine-Resistant Familial Mediterranean Fever: A Randomized, Double-Blind, Placebo-Controlled Trial. Arthritis Rheumatol..

[39] Brik R., Butbul-Aviel Y., Lubin S., Ben Dayan E., Rachmilewitz-Minei T., Tseng L., Hashkes P.J. (2014). Canakinumab for the treatment of children with colchicine-resistant familial Mediterranean fever: A 6-month open-label, single-arm pilot study. Arthritis Rheumatol..

[40] De Benedetti F., Gattorno M., Anton J., Ben-Chetrit E., Frenkel J., Hoffman H.M., Koné-Paut I., Lachmann H.J., Ozen S., Simon A. (2018). Canakinumab for the Treatment of Autoinflammatory Recurrent Fever Syndromes. N. Engl. J. Med..

[41] Hashkes P.J., Spalding S.J., Giannini E.H., Huang B., Johnson A., Park G., Barron K.S., Weisman M.H., Pashinian N., Reiff A.O. (2012). Rilonacept for colchicine-resistant or -intolerant familial Mediterranean fever: A randomized trial. Ann. Intern. Med..

[42] Lachmann H.J., Lauwerys B., Miettunen P., Kallinich T., Jansson A., Rosner I., Manna R., Murias S., Savic S., Smeets S. (2021). Canakinumab improves patient-reported outcomes in children and adults with autoinflammatory recurrent fever syndromes: Results from the CLUSTER trial. Clin. Exp. Rheumatol..

[43] Ozen S., Ben-Cherit E., Foeldvari I., Amarilyo G., Ozdogan H., Vanderschueren S., Marzan K., Kahlenberg J.M., Dekker E., De Benedetti F. (2020). Long-term efficacy and safety of canakinumab in patients with colchicine-resistant familial Mediterranean fever: Results from the randomized phase III CLUSTER trial. Ann. Rheum. Dis..

[44] Henes J.C., Saur S., Kofler D.M., Kedor C., Meisner C., Schuett M., Krusche M., Koetter I., Xenitidis T., Schulze-Koops H. (2022). Tocilizumab for the Treatment of Familial Mediterranean Fever-A Randomized, Double-Blind, Placebo-Controlled Phase II Study. J. Clin. Med..

[45] Koga T., Sato S., Hagimori N., Yamamoto H., Ishimura M., Yasumi T., Kirino Y., Ikeda K., Yachie A., Migita K. (2022). A randomized, double-blind, placebo-controlled phase III trial on the efficacy and safety of tocilizumab in patients with familial Mediterranean fever. Clin. Exp. Rheumatol..

[46] Mertz P., Hentgen V., Georgin-Lavialle S. (2025). Could tocilizumab be used in familial Mediterranean fever? A systematic review. Rheumatology.

[47] Veyssiere M., Sadat Aghamiri S., Hernandez Cervantes A., Henry T., Soumelis V. (2023). A mathematical model of Familial Mediterranean Fever predicts mechanisms controlling inflammation. Clin. Immunol..

[48] Garcia-Robledo J.E., Aragón C.C., Nieto-Aristizabal I., Posso-Osorio I., Cañas C.A., Tobón G.J. (2019). Tofacitinib for familial Mediterranean fever: A new alternative therapy?. Rheumatology.

[49] Karadeniz H., Güler A.A., Atas N., Satış H., Salman R.B., Babaoglu H., Tufan A. (2020). Tofacitinib for the treatment for colchicine-resistant familial Mediterranean fever: Case-based review. Rheumatol. Int..

[50] Gök K., Cengiz G., Erol K., Ozgocmen S. (2017). Tofacitinib suppresses disease activity and febrile attacks in a patient with coexisting rheumatoid arthritis and familial Mediterranean fever. Acta Reumatol. Port..

[51] Piram M., Kone-Paut I., Lachmann H.J., Frenkel J., Ozen S., Kuemmerle-Deschner J., Stojanov S., Simon A., Finetti M., Sormani M.P. (2014). Validation of the auto-inflammatory diseases activity index (AIDAI) for hereditary recurrent fever syndromes. Ann. Rheum. Dis..

[52] Koné-Paut I., Piram M., Benseler S., Kuemmerle-Deschner J.B., Jansson A., Rosner I., Tommasini A., Murias S., Karadag O., Levy J. (2022). Use of the Auto-inflammatory Disease Activity Index to monitor disease activity in patients with colchicine-resistant Familial Mediterranean Fever, Mevalonate Kinase Deficiency, and TRAPS treated with canakinumab. Jt. Bone Spine.

[53] Piskin D., Arici Z.S., Konukbay D., Romano M., Makay B., Ayaz N., Bilginer Y., Berard R.A., Poyrazoglu H., Kasapcopur O. (2022). Number of Episodes Can Be Used as a Disease Activity Measure in Familial Mediterranean Fever. Front. Pediatr..

[54] Demirkaya E., Acikel C., Hashkes P., Gattorno M., Gul A., Ozdogan H., Turker T., Karadag O., Livneh A., Ben-Chetrit E. (2016). Development and initial validation of international severity scoring system for familial Mediterranean fever (ISSF). Ann. Rheum. Dis..

[55] Ter Haar N.M., Annink K.V., Al-Mayouf S.M., Amaryan G., Anton J., Barron K.S., Benseler S.M., Brogan P.A., Cantarini L., Cattalini M. (2017). Development of the autoinflammatory disease damage index (ADDI). Ann. Rheum. Dis..

[56] Ter Haar N.M., van Delft A.L.J., Annink K.V., van Stel H., Al-Mayouf S.M., Amaryan G., Anton J., Barron K.S., Benseler S., Brogan P.A. (2018). In silico validation of the Autoinflammatory Disease Damage Index. Ann. Rheum. Dis..

[57] Ozen S., Demirkaya E., Duzova A., Erdogan O., Erken E., Gul A., Kasapcopur O., Kasifoglu T., Kisacik B., Ozdogan H. (2014). FMF50: A score for assessing outcome in familial Mediterranean fever. Ann. Rheum. Dis..

[58] Yesilkaya S., Acikel C., Fidanci B.E., Polat A., Sozeri B., Ayaz N.A., Makay B.B., Simsek D., Akinci N., Ozcelik G. (2015). Development of a medication adherence scale for familial Mediterranean fever (MASIF) in a cohort of Turkish children. Clin. Exp. Rheumatol..

[59] Koga T., Sato S., Furukawa K., Yamamoto H., Kawakami A. (2025). Investigating the impact of tocilizumab on serum cytokines concentrations in Japanese FMF patients: A sub-analysis of the NUH01FMF study. Immunol. Med..

[60] Ozeri D.J., Bar D., Somech Safran B., Druyan A., Kukuy O.L., Giat E., Lidar M., Livneh A. (2025). Renal outcomes and survival in amyloidosis associated with familial Mediterranean fever: A longitudinal study. Semin. Arthritis Rheum..

[61] Colak S., Tekgoz E., Cinar M., Yilmaz S. (2021). The assessment of tocilizumab therapy on recurrent attacks of patients with familial Mediterranean fever: A retrospective study of 15 patients. Mod. Rheumatol..

[62] Ugurlu S., Hacioglu A., Adibnia Y., Hamuryudan V., Ozdogan H. (2017). Tocilizumab in the treatment of twelve cases with aa amyloidosis secondary to familial mediterranean fever. Orphanet J. Rare Dis..

[63] Cebeci S.O., Yildiz M., Gunalp A., Cebi M.N., Kilinc B., Pinar E., Konte E.K., Aslan E., Haslak F., Adrovic A. (2024). The efficacy of a single-dose anakinra injection during disease attack in pediatric familial Mediterranean fever. Rheumatol. Int..

